# Contemporary strategies for repair of complex thoracoabdominal aortic aneurysms: real-world experiences and multilayer stents as an alternative

**DOI:** 10.1590/1677-5449.011417

**Published:** 2017

**Authors:** Ralf Robert Kolvenbach

**Affiliations:** 1 Catholic Hospital Group Duesseldorf, Department of Vascular Surgery and Endovascular Therapy, Augusta Hospital, Duesseldorf, Germany.

**Keywords:** aortic aneurysm, vascular surgical, endovascular procedures, stents, endoleak, aneurisma de aorta, cirurgia vascular, procedimentos endovasculares, stents, endoleak

## Abstract

Thoracoabdominal aortic aneurysms (TAAA) present special challenges for repair due to their extent, their distinctive pathology, and the fact that they typically cross the ostia of one or more visceral branch vessels. Historically, the established treatment for TAAA was open surgical repair, with the first procedure reported in 1955. Endovascular repair of TAAA with fenestrated and/ or branched endografts, has been studied since the beginning of the current century as a means of mechanical aneurysm exclusion. More recently, flow modulator stents have been employed with the aim at reducing shear stress on aortic aneurysmal wall. In this review we present technical and main results of these techniques, based on literature review and personal experience.

## Resumo

Aneurismas da aorta toracoabdominal apresentam desafios especiais no seu reparo devido à sua extensão, patologia distinta, e pelo fato de que tipicamente eles atravessam o óstio de um ou mais vasos de ramos viscerais. Historicamente, o tratamento estabelecido para aneurismas da aorta toracoabdominal foi o reparo em cirurgia aberta, com o primeiro procedimento relatado em 1955. O reparo endovascular de aneurismas da aorta toracoabdominal com endoenxertos fenestrados e/ou ramificados tem sido estudado desde o início deste século como meio de exclusão mecânica do aneurisma. Mais recentemente, stents moduladores de fluxo têm sido empregados com o objetivo de reduzir o estresse de cisalhamento na parede do aneurisma de aorta. Nesta revisão, apresentamos os principais resultados obtidos com essas técnicas, com base em revisão de literatura e experiência pessoal.

## INTRODUCTION

Thoracoabdominal aortic aneurysms (TAAA) present special challenges for repair due to their extent, their distinctive pathology, and the fact that they typically cross the ostia of one or more visceral branch vessels. TAAA patients frequently have significant coexisting medical conditions – including hypertension (a prevalence of ≥ 80% at baseline in most clinical trials of TAAA repair), coronary artery disease, chronic obstructive pulmonary disease (COPD), congestive heart failure, cerebrovascular occlusive disease, and peripheral arterial disease – that can reduce their fitness for surgery, increasing the risk of peri- and post-procedural mortality and serious morbidity.^1^^,^ 2 Historically, the established treatment for TAAA was open surgical repair, with the first procedure reported in 1955.^3^ Endovascular repair of TAAA with fenestrated and/or branched endografts, which has been studied since the beginning of the current century as a means of mechanical aneurysm exclusion,^4^^,^ 5 evolved from the practice of endovascular repair of TAA, which was pioneered in the mid 1990s.^6^ The more recently developed Multilayer Flow Modulator (MFM) (Cardiatis, Isnes, Belgium) is a distinct endovascular intervention strategy that employs physiological and hemodynamic principles with the aim of reducing shear stress on the aortic wall, stabilizing the aneurysm, and laminating blood flow.

## OPEN REPAIR OF TAAA

Beginning in the 1980s, classical open repair of TAAA included a clamp-and-sew approach (usually without distal perfusion), routine cerebrospinal fluid (CSF) drainage, aggressive intercostal reimplantation, regional hypothermia for spinal cord protection, infusion of hypothermic renal preservation fluid, and in-line mesenteric shunting.^7^ Although this open-repair approach was found over time to be generally effective and durable in treating aneurysms and preventing rupture, it also involved serious risks of early mortality and morbidity.^8^^,^ 9 A modified surgical approach for type I to type III TAAA – sometimes referred to as the collateral network concept – includes routine use of distal aortic perfusion via left atrial to femoral bypass, motor evoked potential monitoring, and only selective intercostal reimplantation as indicated.^7^^,^ 10 The key issues in open repair of TAAA are related to protection of the lower body organs while the aorta is cross-clamped and to the methods to be used for reattaching the visceral arteries (SMA, celiac axis, and renal arteries).^11^

In addition to perioperative mortality, the serious complications most commonly associated with open surgical repair of TAAA include renal failure, spinal cord ischemia (SCI), COPD, stroke, and myocardial infarction (MI). Depending in part on definitional criteria, the reported incidence of renal dysfunction after open TAAA repair ranges from 4% to 40%.^10^^,^ 12 The pathophysiology of the renal dysfunction is understood to be multifactorial, but including the release of cytokines and other inflammatory mediators resulting from ischemia during aortic cross-clamping above the mesenteric and renal arteries.^1^ SCI is usually diagnosed within the first postoperative day and almost always within the first week, with the etiology including elevation of intrathecal pressure and spinal hypoperfusion, with the viability of spinal cord cells dependent on arteries arising from the low intercostal or lumbar territory that may be temporarily or permanently excluded during TAAA surgery. Current estimates of the incidence of SCI after open TAAA repair range from 2 to 20%.^1^^,^ 13 ^,^ 14 Because type I and type II TAAA involve most of the descending thoracic aorta, they are associated with the greatest risk for SCI – odds ratios of 27 for type I and 39 for type II.^15^

Reporting of pulmonary complications, which are common after open repair of TAAA, is also subject to variation in terms of categorization.^1^ In a study of pulmonary complications in 219 cases of open surgical repair for TAAA (n = 140) and TAA (n = 79), adverse perioperative outcomes included hospital death in 21 (5.9%), stroke in 13 (5.9%, 5 of whom died), and respiratory complications with prolonged postoperative ventilation in 60 (27%), with 24 (11%) requiring tracheostomy.^16^ It is said that the only relatively surefire strategies for preventing lung complications during TAAA repair are the avoidance of incisions that might lead to pain-induced respiratory dysfunction and the elimination of general anesthesia – conditions that can potentially be met with endovascular repair.^1^

A long-standing benchmark for morbidity and mortality in open TAAA surgery was established by the 1993 report of experience with 1509 patients at the pioneering Baylor College of Medicine (Houston, Texas) – including a 30-day survival rate of 92%, a 16% incidence of SCI (paraplegia or paraparesis), and kidney failure occurring in 18% of patients (9% requiring dialysis).^17^ However, an analysis on the US Nationwide Inpatient Sample database from 1988 to 1998 found a 22.3% rate of operative mortality for elective open repair of TAAA in 1542 cases, with overall postoperative complication rates over 50%.^18^ In a meta-analysis of 7833 cases of open repair of TAAA performed between 2000 and 2010, the rate of 30-day mortality was 7%, SCI was 7.5%, renal failure was 19%, and pulmonary dysfunction was 36%.^19^

The incidence of the surgical complications has decreased over time along with the improvements in open surgical approach, with the best rates achieved at experienced high-volume centers. Table 1 summarizes the short-term and long-term experience with open surgical repair of TAAA at two such centers – Baylor College of Medicine (Houston, Texas) and St. Antonius Hospital (Nieuwegein, Netherlands). At Baylor, Coselli et al. reported on 3264 repair procedures performed between 1986 and 2014.^20^ They recorded an overall perioperative adverse event rate of 14.4% and noted that the rate of adverse events was highest in repair of type II TAAA (203/1066, 19.0%) and lowest in repair of type IV TAAA (67/669, 10.0%). Independent predictors of early mortality were increasing age, renal dysfunction, type II and IV TAAA, rupture, involvement of visceral vessels, and increasing clamp time. Estimated postoperative survival was 83.5%, 63.6%, 36.8%, and 18.3% at 1, 5, 10, and 15 years. Of 88 late repair failures, 44 underwent aortic reinterventions; 29 of the 88 patients with repair failure remained alive.^20^

**Table 1:** Open repair of TAAA – perioperative and long-term outcomes from two high-volume centers.

Outcomes	Coselli et al. (Baylor)^20^ n = 3309	Murana et al. (St. Antonius)^10^ n = 542
Perioperative outcomes (≤ 30 days)		
Crawford type	I, 914; II, 1066; III, 660; IV, 669	I, 128; II, 285; III, 62; IV, 48; V, 19
Operative death (≤ 30 days)	249 (7.5%)	46 (8.5%)
Spinal cord ischemia	178 (5.4%)	32 (5.9%)
Renal failure necessitating dialysis	250 (7.6%)	23 (4.2%)
Stroke	98 (3.0%)	23 (4.2%)
Respiratory failure	281 (8.5%)	42 (7.7%)
Myocardial infarction	41 (1.2%)	13 (2.4%)
Long-term outcomes		
Mean follow-up		6.32 years
Freedom from repair failure	5 years: 97.9%±0.3% 10 years: 95.3%±0.6% 15 years: 94.1%±0.8%	
Freedom from reintervention		1 year: 96.1%±0.1% 5 years: 86.3%±1.8% 10 years: 80.7%±2.3%
Estimated survival	1 year: 83.5%±0.7% 5 years: 63.6%±0.9% 10 years: 36.8%±1.0% 15 years: 18.3%±0.9%	1 year: 85.9%±1.5% 5 years: 74.2%±2.0% 10 years: 61.6%±2.5%

At St. Antonius Hospital, Murana et al. reported on 542 open TAAA repairs performed between 1994 and 2014.^10^ Independent predictors of 30-day mortality, which was 8.5%, were age, female gender, and urgent and emergency versus elective repair. Estimated postoperative survival was 85.9%, 74.2%, and 61.6% at 1, 5, and 10 years, with aortic reinterventions required by 8.5% of the patients.^10^

## ENDOVASCULAR REPAIR OF TAAA WITH FENESTRATED AND/OR BRANCHED ENDOGRAFTS

Compared to open surgery, endovascular repair of TAAA avoids major thoracoabdominal incisions and aortic cross-clamping and allows limitation of blood loss, perioperative pain, and respirator dependency, potentially reducing the incidence of visceral, renal, and spinal cord ischemia.^15^ Endovascular repair of aneurysms in the thoracic aorta has extended treatment applicability to many patients who would be unfit for open surgery while at the same time significantly reducing periprocedural and short-term mortality and yielding comparable midterm outcomes.^21^^,^ 22 At the same time, significant costs and long manufacturing delays are involved with the device customization for TAAA repair, and the need for advanced endovascular experience and skills has meant that optimal outcomes are only achievable at high-volume specialist centers.

### Adapting endograft technology for treatment of TAAA

Endovascular treatment of TAAA entails the challenge of preserving the branch vessels, particularly any visceral and renal arteries that will be covered by the endograft. The common strategy has been to create modular endografts with configurations that allow openings to and connections with the “target” branch vessels that will be involved.^4^ Fenestrated endografts are constructed with reinforced fenestrations (window openings), through which balloon-expandable or self-expanding covered (“bridging”) stents can be extended into target branch vessels – with the proximal end of the covered stent flared at the fenestration to create a seal. Sometimes the fenestrations are scalloped openings in the proximal or distal edge of the endograft fabric that are designed to allow incorporation of segments of the visceral arteries into the proximal or distal sealing zones. As an alternative to the construction with fenestrations, the endografts can be custom-made with dedicated side-branch cuffs directly attached. From the cuffs, balloon-expandable or self-expanding covered bridging stents can then be extended into the target branch vessels. The covered stents deployed in the side branches in fenestrated endovascular aortic repair (FEVAR) or branched endovascular aortic repair (BEVAR) can be further reinforced with self-expanding or balloon-expandable bare metal (“re-lining”) stents to prevent kinking.^23^ Fenestrations are generally preferred for right-angle take-off of visceral arteries; side-branch cuffs are usually preferred with larger aortic diameters and when target branch vessels have a downward path.^24^ In many cases of endovascular treatment of complex TAAA, the endografts that are employed include both fenestrations and branches.^24^

Fenestrated endografts were initially used to treat juxtarenal aortic aneurysms (JRAA) with short infrarenal necks.^25^^,^ 26 With the development of bridging stent graft technology, the use of fenestrated endografts was extended to cover pararenal aortic aneurysms (PRAA) and type IV TAAA and eventually types I, II, and III TAAA.^27^^,^ 28 Fenestrated and/or branched endografts for the treatment of TAAA are currently sourced in three different ways. (1) Sometimes, physicians will themselves modify existing TAA or AAA endografts, making fenestrations and/or adding side-branch cuffs specific to the anatomy of particular TAAA patients. (2) Some device companies market endografts that can be custom-manufactured with fenestrations and/or cuffs based on individual patient measurements from high-resolution CT scans of the entire chest, abdomen, and pelvis, with the manufacture requiring as long as 10 weeks. (3) Most recently, for “off-the-shelf” use, some companies are manufacturing endografts with prefabricated fenestrations and/or branch cuffs that may be suitable for the anatomical distribution of renal and visceral branch vessels in 60% to 80% of cases.^29^

### Outcomes of fenestrated and/or branched endovascular repair of TAAA

Outcomes in studies of fenestrated and/or branched endovascular repair include early all-cause mortality and morbidity rates calculated perioperatively and up to and including postoperative day 30. At and after 1 year, mortality and morbidity are commonly assessed by Kaplan-Meier life-table analysis. Technical/procedural success is conventionally defined as successful deployment of the endograft in the intended anatomic position and completion of the procedure with no type I or III endoleaks and without the need for a secondary intervention (including conversion to open surgery) within a defined time period. In addition to these endpoints, reported complications include device migration (also unique to endovascular repair), branch vessel occlusion, rupture, aortic dissection, SCI, renal failure, respiratory failure, and MI. Performance assessments can include branch vessel patency and change in aneurysm diameter.

Rates reported for type I and III endoleak in studies of endovascular TAAA repair generally range from 0 to 20%.^30^ As with open repair of TAAA, the postoperative complications of greatest concern in endovascular repair are renal failure and SCI. The reported incidence of postoperative renal insufficiency ranges as high as 33% in studies of endovascular TAAA repair.^31^ Postoperative renal impairment has been associated with comorbid peripheral arterial disease, long-lasting procedures, repair of complex and extensive TAAA, and the presence of thrombus at the level of the visceral arteries.^32^ Renal injury after TAAA repair with relatively stiff and noncompliant fenestrated and/or branched covered stent grafts has been associated with a postimplantation systemic inflammatory reaction involving leukocytosis and thrombocytopenia.^33^ Current estimates of the incidence of SCI after endovascular TAAA repair range as high as 30%.^1^^,^ 15 ^,^ 34 ^-^ 36 As with open repair, type I and type II TAAA are associated with the greatest risk for SCI – odds ratios of 20 for type I and 14 for type II.^15^ The incidence of SCI has been linked to the extent of coverage of intercostal and lumbar arteries and as well to the duration of the procedure. Measures employed for reducing the risk of SCI during fenestrated and/or branched endovascular TAAA repair – aimed at preserving perfusion by augmenting cardiac function and reducing CSF pressure – include CSF drainage (in patients with type I, II, and III TAAA), use of a temporary perfusion branch (to maintain blood circulation in the aneurysm sac for a period of time), and staging of the entire procedure so that the insertion of bridging covered stents to the visceral arteries is performed secondarily, a few days after the initial placement of the main aortic endograft.^23^^,^ 24 ^,^ 32

Table 2 summarizes short-term and midterm experience with fenestrated and/or branched repair of TAAA at two leading centers (one in the United States,^26^ one in Germany^24^) and in a multicenter French trial.^32^ At the Cleveland Clinic, Eagleton et al. evaluated commercially customized fenestrated and/or branched endografts for 354 high-surgical-risk patients with extensive type II and III TAAA.^26^ The endografts had 1305 fenestration/branches. Perioperative mortality was greater in repairs of type II TAAA compared to type III TAAA (7.0% vs 3.5%, p < 0.001). SCI developed in 21 (16.4%) patients with type II TAAA but only 10 (4.4%) with type III TAAA (p < 0.001); permanent SCI occurred in 10 (7.8%) patients with type II TAAA and 4 (1.8%) with type III TAAA (p = 0.005). Reinterventions were required in 27 branch vessels (7.6%) for stenosis or occlusion; 80 endoleak repairs were performed in 67 patients, including 55 branch-related endoleaks. Factors negatively affecting survival were the presence of type II TAAA (p < 0.01), older age (p < 0.01), and COPD (p < 0.05).

**Table 2:** Fenestrated and/or branched repair of TAAA – perioperative and midterm outcomes from two leading centers and a multicenter trial.

Outcomes	Verhoeven et al. (Paracelsus)^24^ n = 166	Eagleton et al. (Cleveland Clinic)^26^ n = 354	Marzelle et al. (WINDOWS trial)^32^ n = 268
Perioperative outcomes (≤ 30 days)			
Crawford type	I, 12; II, 50; III, 53; IV, 41; V, 10	II, 128; III, 226	I, 2; II 16; III, 24; IV, 26
Target branch arteries	600	1305	1463
Technical success	157 (95%)	333 (94.1%)	230/252 (91.2%)
Operative death (≤30 days)	13 (7.8%)	17 (4.8%)	18 (6.7%)
Spinal cord ischemia	15 (9%)	31 (8.8%)	11 (4.1%)
Renal failure necessitating dialysis	9 (5.4%)	10 (2.8%)	15 (5.6%)
Stroke	2 (1.2%)	8 (2.3%)	5 (1.9%)
Respiratory failure	6 (3.6%)	32 (9.0%)	14 (5.2%)
Myocardial infarction	9 (5.4%)	10 (2.8%)	4 (1.5%)
Branch vessel occlusion	2 (1.2%)	4 (1.1%)	8/252 (3.2%)
Rupture	1 (0.6%)	1 (0.3%)	1 (0.4%)
Type I/III endoleak		10 (2.8%)	15 (5.6%)
Early reintervention	12 (7.2%)	13 (3.7%)	31 (11.6%)
Long-term outcomes			
Mean follow-up	29.2±21 months	22±19 months	
Estimated target branch vessel patency	1 year: 98%±0.6% 2 years: 97%±0.8% 5 years: 94.2%±1.5%	3 years: CA 96% (95%CI 0.93-0.99); SMA 98% (95%CI 0.97-1.0); RA 98% (95%CI 0.96-1.0)	
Reintervention for endoleak	20 (12.0%)	67 (18.9%)	
Freedom from reintervention	1 year: 88.3%±2.7% 3 years: 78.4%±4.5%	3 years: 54% (95%CI 0.47-0.61)	
Estimated survival	1 year: 83%±3% 2 years: 78%±3.5% 5 years: 66.6%±6.1%	3 years: 57% (95%CI 0.50-0.63)	

At Paracelsus Medical University in Nurnberg, Germany, Verhoeven et al. evaluated their 10-year experience with customized fenestrated and/or branched endografts for 166 TAAA patients, 108 (65%) of whom had been refused for open surgery.^24^ Coverage was planned for 600 visceral and renal branch arteries – with 274 fenestrations and 326 branch cuffs. The 30-day operative mortality was 7.8%, and the in-hospital mortality was 9%. Perioperative SCI occurred in 9% of patients, and permanent paraplegia in 1.2% of patients. During a mean follow up of 29.2±21 months, 40 patients died. Aneurysm sac shrinkage was noted in 69% of patients, no significant change in 26%, and sac expansion in 5%. Estimated freedom from sac expansion at 1, 3, and 5 years was 99.3%, 94.3%, and 83.2%, respectively. Late reintervention (> 30 days) was required in 28 patients – a total of 36 events, including target vessel bridging stent relining or extension (for endoleak or stenosis) in 18 cases.

The prospective WINDOWS trial reported early outcomes for the use of customized fenestrated and/or branched stent-grafts in 268 patients with juxtarenal and pararenal AAA, suprarenal aneurysms, and TAAA conducted at 8 university hospitals in France from 2009 to 2012.^32^ The patients were at high risk for open surgery, with a mean number of 3.2±1.6 risk factors. The overall rate of technical success was 91.2% (blood transfusions required in 43.3%); 30-day mortality was 6.7%, in-hospital mortality was 10.1%, and the 30-day rate of combined mortality and severe complications was 22.0%. Complications included severe renal insufficiency in 5.6% and aneurysm-related reintervention in 11.6%. SCI developed in 11 (4.1%) patients overall and in 7 (16.6%) of the 42 patients with type I (n = 2), type II (n = 16), or type III (n = 24) TAAA (hazard ratio 15.96). SCI was associated with in-hospital mortality with a hazard ratio of 9.46. As the most frequent cause of death was multiorgan failure, the authors noted the need for investigation into the role of inflammatory response to the exclusion in large aortic segments.^32^

## THE MFM ALTERNATIVE FOR TREATMENT OF TAAA

A distinct endovascular intervention strategy for TAAA repair is implantation of the Multilayer Flow Modulator (MFM) (Cardiatis, Isnes, Belgium). The MFM is an off-the-shelf uncovered self-expanding stent with three-dimensional wire layering designed to modulate blood flow dynamics to thrombose, stabilize, and support remodeling of the aneurysm sac, while reducing shear stress on the aortic wall and buffering against the risk of rupture at the most vulnerable points.^37^^,^ 38 In contrast with the relative stiffness and noncompliance of covered stent grafts, the open architecture of the MFM can promote more rapid and complete re-endothelialization and integration with and healing recovery of the vessel wall, limiting the potential for the postimplantation systemic inflammatory reaction noted after fenestrated and/or branched endograft repair.^33^^,^ 39 As blood flows through the wire layering and exits at the outermost layer of the device, it is organized into a laminar flow channel for perfusion of branch vessels, without the need for the extra steps involved in cannulation and placement of bridging stent grafts (with any pre-existing occlusion or stenosis in the branch vessels having been treated before the MFM implantation).^37^ Where there is no branch involvement, with the elimination of the dynamic shear vortex within the aneurysm, the flow is redirected along the aortic wall in the same direction as the systemic pressure. Thus as a consequence of the flow modulation promoted by the porosity of the three-dimensional braided mesh, perfusion can be more readily maintained for the visceral and renal arteries and the spinal cord, and the potential for renal function impairment and SCI can be greatly reduced.

### Initial outcomes with MFM treatment of TAAA

Among studies of MFM treatment for TAAA are a prospective multicenter French trial,^40^^,^ 41 a prospective single-center Moroccan registry,^42^ and an independent global MFM registry.^43^^,^ 44 Table 3 summarizes perioperative, midterm, and (in the multicenter trial) long-term outcomes for the patients in these studies. These patients had extensive aortic pathology and significant comorbidities, all were considered to be at high surgical risk (most were ASA class 3 or 4), and many were also contraindicated for fenestrated and/or branched endograft repair. The MFM treatment in these patients involved coverage of multiple branch vessels. Notwithstanding the relatively small numbers of patients in these initial studies with the MFM, the outcomes summarized in Table 3 compare favorably with those for open and fenestrated and/or branched repair of TAAA – high rates of technical success and branch vessel patency, with very limited rates of the serious complications that have been most prevalent following treatment with the other modalities.

**Table 3:** Multilayer flow modulator repair of TAAA **–** outcomes from two prospective trials and a retrospective registry review.

Outcomes	Vaislic et al. (STRATO multicenter)^40^^,^ 41 n = 23	Benjelloun et al. (single-center Moroccan registry)^42^ n = 18	Sultan et al. (initial MFM patients in 12 countries)^43^^,^ 44 n = 103
Perioperative outcomes (≤ 30 days)			
Indications	TAAA: II, 10; III, 13	TAAA: I, 4; II, 2; IV, 4; AAA: 8	TAAA: I, 11; II, 14; III, 26; IV, 24; arch aneurysms: 7; suprarenal AAA: 15; type B dissections: 6
Target branch arteries	55	61	378
Technical success	23 (100%)	18 (100%)	100 (97.1%)
Operative death (≤30 days)	0 (0%)	0 (0%)	0 (0%)
Spinal cord ischemia	0 (0%)	0 (0%)	1 (0.99%)^*^
Renal failure necessitating dialysis	0 (0%)	0 (0%)	0 (0%)
Stroke	0 (0%)	0 (0%)	0 (0%)
Respiratory failure	0 (0%)	0 (0%)	
Myocardial infarction	0 (0%)	0 (0%)	
Branch vessel occlusion	2/55 (3.6%)	0 (0%)	0 (0%)
Rupture	0 (0%)	0 (0%)	0 (0%)
Type I/III endoleak	1 (4%)	0 (0%)	
Early reintervention	1 (4%)	0 (0%)	2 (1.94%)^*^
Long-term outcomes			
Mean follow-up		13.4 months	11.6±3.3 months
Target branch vessel patency	1 year: 100% 2 years: 100% 3 years: 96.6% 4 years: 100%	1 year: 100%	1 year: 95.3%
Reintervention for endoleak	10 (43.5%)	1 (5.5%)	
Freedom from reintervention			1 year: 89.3%
Cumulative mortality	1 year: 1 (4.3%) 2 years: 3 (13.0%) 3 years: 8 (34.8%) 4 years: 11 (47.8%)	1 year: 3 (16.7%)	
Estimated survival			1 year: 86.8%

* One successful deployment of a second MFM within the first 30 days, to correct for device retraction into the aneurysm sac caused by stent foreshortening; one conversion to open repair at 30 days to correct for proximal device infolding, the conversion complicated by postoperative paraplegia.

Outcome data are available out to 4 years for the STRATO trial, in which 23 patients (mean age 75.8 years, 19 men) with type II (43.5%) and III (56.5%) TAAA (mean diameter 6.5 cm) were treated between April 2010 and February 2011 at 10 centers in France.^40^^,^ 41 Patient comorbidities included hypertension in 87%, peripheral artery disease in 56.5%, and coronary artery disease in 26.1%, and 65.2% had undergone previous aortic interventions. There was no in-hospital or 30-day mortality; none of the 11 deaths that occurred through 4 years of follow-up were confirmed as being aneurysm related. Through 4 years, there were no reported cases of SCI, confirmed aneurysm rupture, device migration or fracture, or respiratory, renal, or peripheral complications (Table 3). Through 4 years, reinterventions were performed for 11 patients (for type I or III endoleak in 10). Patency was achieved for 96.4% (53/55) of target branch vessels at 1 year; secondary patency at 1 year was 100% after successful surgical intervention on 2 occluded vessels in 1 patient who was not given dual antiplatelet therapy but only aspirin. Patency was then 100% (32/32) at 2 years, 96.6% (28/29) at 3 years, and 100% (9/9) at 4 years. Aneurysm sac thrombosis and successful reduction of residual aneurysm flow was achieved for 75.0% (15/20) at 1 year, 92.3% (12/13) at 2 years, 90.9% (10/11) at 3 years, and 75.0% (3/4) at 4 years.

In the Moroccan registry, 18 patients (mean age 61.1 years, 16 men) with TAAA (n = 10, mean diameter 74.4 mm) and AAA (n = 8, mean diameter 67.8 mm) were treated with the MFM between June 2009 and September 2012.^42^ The AAA were aorto-bi-iliac in 6 patients and juxtarenal in 5. Patient comorbidities included 9 with coronary artery disease, 7 with diabetes mellitus, and 6 each with respiratory insufficiency, hypertension, dyslipidemia, and peripheral artery disease. Two of the patients entered the study with surgical interventions prescheduled for peripheral artery disease. During mean follow-up of 13.4 months, 3 patients died, with the cause of death unrelated in 2 and undetermined in 1. There were no cases of SCI, rupture, or device migration, kinking, or fracture, and there was no renal impairment associated with the MFM implantation (Table 3). The only reintervention was implantation of an additional MFM device at 5 years after the index procedure in a young patient with a type I endoleak that was considered to be possibly due to natural growth of the aorta. No other endoleaks were observed. All 61 covered branch vessels remained patent through the follow-up. Among 9 TAAA patients with at least 6 months follow-up, aneurysm thrombosis was complete in 4 and partial in 5 (with residual aneurysm flow approximately 25% in 3, 65% in 1, and 75% in 1). Aneurysm thrombosis was complete for all 6 AAA patients with at least 6 months follow-up.

In 2014 Sultan et al. reported early midterm outcomes for the first 103 patients treated under the device instructions for use (IFU) beginning in August 2010 in a global MFM registry comprising 380 cases overall.^43^^,^ 44 The indications in the 103 patients (mean age 69.2 years), who were treated on a compassionate basis, included 75 TAAA, 7 arch aneurysms, 15 suprarenal AAA, and 6 type B dissections (Table 3). There was no 30-day mortality or visceral or renal insult. Through mean follow-up of 11.6±3.31 months, there was no aneurysm rupture and no stent fracture. There were 2 cases (1.9%) of hemorrhagic stroke, both resulting in death. The rate of SCI was 0.99%, with the only case occurring at 30 days as a complication of surgical conversion to correct for proximal infolding of the index MFM. There were 11 endovascular reinterventions, all of which involved successful implantation of an additional MFM to correct for retraction of the index MFM back into the aneurysm sac due to stent foreshortening, and 1 surgical reintervention to treat progression of atherosclerotic disease distal to the MFM implantation. At 1 year, 95.3% of 378 target branch vessels were patent. Overall, aneurysm sac remodeling was demonstrated as the ratio of thrombus to total volume stayed almost constant over 1 year while the ratio of aneurysm flow volume to total volume fell.

### Important considerations in MFM treatment of TAAA

It is useful to note that because the MFM is not a covered stent but rather permits porosity in the range of 65%, the only endoleak categories that apply to its use are types I (failure mode I, due to incomplete or ineffective sealing at either the proximal or distal end of the stented segment) and III (failure mode II, due to inadequate overlapping of multiple devices). As consistently noted in the published reports on the initial MFM studies that are summarized here, most of the type I and III endoleaks detected periprocedurally and during follow-up were adjudicated as being due to poor compliance with the device IFU — in terms of the adequacy of proximal or distal device landing zones and overlap zones or the correct procedure for overlapping devices (the smaller device should be deployed before the bigger one in an overlap situation).^40^^-^ 42 Endoleaks as well as instances of device migration were also due to the failure during implantation to take into account the potential for foreshortening due to the interwoven design of the MFM and to perform the implantation at a slow enough pace to allow the device to achieve its natural compliance.^42^^-^ 46 Data have not yet been published on outcomes with a new generation of the MFM with flared ends designed to promote aortic wall adherence and reduce the risk of leak at the proximal and distal landing zones.

The point that there is clear potential for improvement in the promising initial results with the MFM by way of more thoroughgoing compliance with the IFU is underscored by a subgroup analysis from the global MFM registry^45^ (38 patients treated outside the IFU) and a systematic review of 15 articles covering 171 MFM patients.^46^ The systematic review identified a total of 39 patients treated outside the IFU, 10 of whom had presented with the contraindication of rupture. For these 39 patients, 1-year aneurysm-related survival was 38.0%±9.0%, compared to 93.3%±2.79% for the 132 patients treated within the IFU (p < 0.001).^46^

Overall in the systematic review, in a total of 449 target branch vessels, the patency rate was 97.8%.^46^ Regarding the other key MFM performance endpoint, complete or partial aneurysm thrombosis was reported in 68 cases in the systematic review, all cases performed within the IFU, whereas there was sac expansion with no stabilization or shrinkage in all of the 38 cases performed outside the IFU in the registry substudy.^45^

Studies with the MFM reported to date, then, suggest that the device can be considered safe and effective when used in compliance with the IFU. Of note in the initial MFM studies under consideration is the near perfect achievement of target branch vessel patency and the near complete absence of complications such as rupture, SCI, and renal failure.^46^ Of course, longer-term trials in larger populations will be required to fully establish the MFM alternative for TAAA repair, as is the case for the newer off-the-shelf fenestrated and/or branched technologies.

### Two cases of TAAA repair with the MFM

#### One-year follow-up of a type II TAAA treated with the MFM

**Figure 1:**
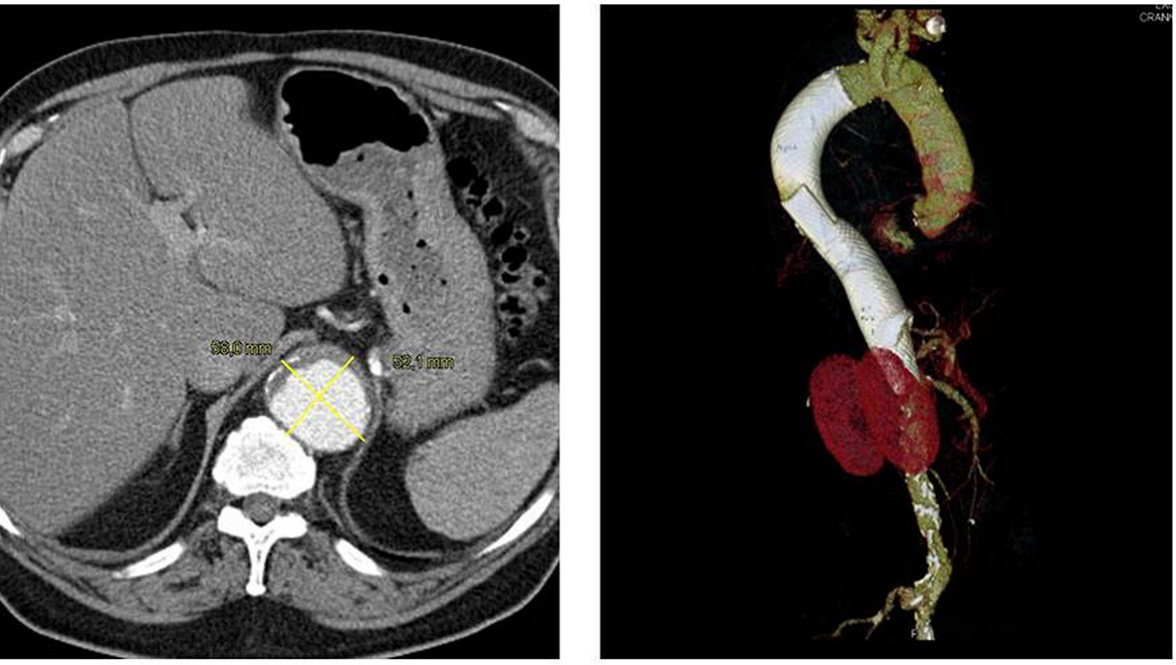
71 year old patient with a thoraco abdominal aortic aneurysm Crawford II classification. Patient was asymptomatic and on Coumadin Therapy for atrial fibrillation. The control CT scans after 1 year show a stable aneurysm diameter although because of the anticoagulation therapy there was no thrombus formation. All visceral branches and both renal arteries were covered by 2 MFM Multilayer stents each 150 cm in length. All branches remained patent during follow up. The patient is still on dual antiplatelet therapy.

A 71-year-old patient presenting with a type II TAAA was asymptomatic. The patient was on Coumadin therapy for atrial fibrillation. The patient was implanted with two MFM devices each 150 cm in length, covering all visceral branch arteries and both renal arteries. Control CT scans 1 year after the MFM implantation (Figure 1) showed a stable aneurysm diameter, although because of the anticoagulation therapy, there was no thrombus formation. All covered branch arteries remained patent during follow-up. The patient remained on dual antiplatelet therapy.

### Three-year follow-up of an 8-cm Taaa TreaTed wiTh 4 mfm devices

**Figure 2:**
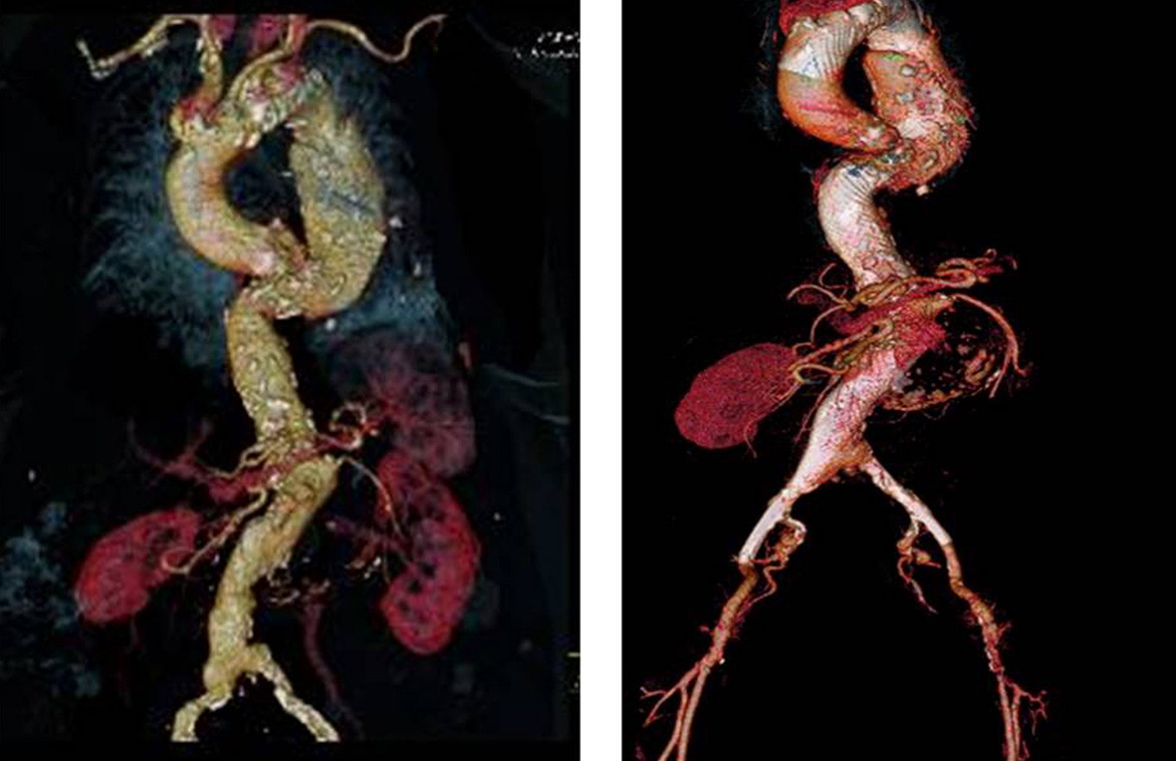
84 year old female patient with a 8 cm in diameter thoraco abdominal aortic aneurysm. The aneurysm increased 6 mm in diameter after one year. After 3 years there was a diameter reduction to 7.5 cm and a stable diameter after that. In this case 4 stents 150 cm to 200 cm in length were implanted with coverage of all renal arteries and visceral branches. The branches have remained patent with the patient on aspirin antiplatelet monotherapy.

An 84-year-old female patient presented with an 8-cm TAAA (Figure 2). The patient was implanted with four MFM devices each 150 cm to 200 cm in length, covering all visceral branch arteries and both renal arteries. At 1-year follow-up, the aneurysm had increased 6 mm in diameter. At 3 years, the aneurysm diameter had been reduced to 7.5 cm (Figure 2B); the diameter then remained stable. The branch arteries remained patent during follow-up, with the patient on antiplatelet monotherapy with aspirin.

## References

[1] Escobar GA, Upchurch GR (2011). Management of thoracoabdominal aortic aneurysms. Curr Probl Surg.

[2] Hansen PA, Richards JM, Tambyraja AL, Khan LR, Chalmers RT (2010). Natural history of thoraco-abdominal aneurysm in high-risk patients. Eur J Vasc Endovasc Surg.

[3] Etheredge SN, Yee J, Smith JV, Schonberger S, Goldman MJ (1955). Successful resection of a large aneurysm of the upper abdominal aorta and replacement with homograft. Surgery.

[4] Greenberg R, Eagleton M, Mastracci T (2010). Branched endografts for thoracoabdominal aneurysms. J Thorac Cardiovasc Surg.

[5] Chuter TA, Gordon RL, Reilly LM, Pak LK, Messina LM (2001). Multi-branched stent-graft for type III thoracoabdominal aortic aneurysm. J Vasc Interv Radiol.

[6] Dake MD, Miller DC, Semba CP, Mitchell RS, Walker PJ, Liddell RP (1994). Transluminal placement of endovascular stent-grafts for the treatment of descending thoracic aortic aneurysms. N Engl J Med.

[7] Patel VI, Lancaster RT, Conrad MF, Cambria RP (2012). Open surgical repair of thoracoabdominal aneurysms - the Massachusetts General Hospital experience. Ann Cardiothorac Surg.

[8] Conrad MF, Crawford RS, Davison JK, Cambria RP (2007). Thoracoabdominal aneurysm repair: a 20-year perspective. Ann Thorac Surg.

[9] Conrad MF, Ergul EA, Patel VI (2011). Evolution of operative strategies in open thoracoabdominal aneurysm repair. J Vasc Surg.

[10] Murana G, Castrovinci S, Kloppenburg G (2016). Open thoracoabdominal aortic aneurysm repair in the modern era: results from a 20-year single-centre experience. Eur J Cardiothorac Surg.

[11] Elefteriades JA, Botta DM (2009). Indications for the treatment of thoracic aortic aneurysms. Surg Clin North Am.

[12] Johns N, Jamieson RW, Ceresa C (2014). Contemporary outcomes of open repair of thoracoabdominal aortic aneurysm in young patients. J Cardiothorac Surg.

[13] Patel VI, Ergul E, Conrad MF (2011). Continued favorable results with open surgical repair of type IV thoracoabdominal aortic aneurysms. J Vasc Surg.

[14] Ferrer C, Cao P, De Rango P (2016). A propensity-matched comparison for endovascular and open repair of thoracoabdominal aortic aneurysms. J Vasc Surg.

[15] Greenberg RK, Lu Q, Roselli EE (2008). Contemporary analysis of descending thoracic and thoracoabdominal aneurysm repair: a comparison of endovascular and open techniques. Circulation.

[16] Etz CD, Di Luozzo G, Bello R (2007). Pulmonary complications after descending thoracic and thoracoabdominal aortic aneurysm repair: predictors, prevention, and treatment. Ann Thorac Surg.

[17] Svensson LG, Crawford ES, Hess KR, Coselli JS, Safi HJ (1993). Experience with 1509 patients undergoing thoracoabdominal aortic operations. J Vasc Surg.

[18] Cowan JA, Dimick JB, Henke PK, Huber TS, Stanley JC, Upchurch GR (2003). Surgical treatment of intact thoracoabdominal aortic aneurysms in the United States: hospital and surgeon volume-related outcomes. J Vasc Surg.

[19] Piazza M, Ricotta JJ (2012). Open surgical repair of thoracoabdominal aortic aneurysms. Ann Vasc Surg.

[20] Coselli JS, LeMaire SA, Preventza O (2016). Outcomes of 3309 thoracoabdominal aortic aneurysm repairs. J Thorac Cardiovasc Surg.

[21] Leurs LJ, Bell R, Degrieck Y, Thomas S, Hobo R, Lundbom J, EUROSTAR, UK Thoracic Endograft Registry collaborators (2004). Endovascular treatment of thoracic aortic diseases: combined experience from the EUROSTAR and United Kingdom Thoracic Endograft registries. J Vasc Surg.

[22] Gopaldas RR, Huh J, Dao TK (2010). Superior nationwide outcomes of endovascular versus open repair for isolated descending thoracic aortic aneurysm in 11,669 patients. J Thorac Cardiovasc Surg.

[23] Oikonomou K, Kopp R, Katsargyris A, Pfister K, Verhoeven EL, Kasprzak P (2014). Outcomes of fenestrated/branched endografting in post-dissection thoracoabdominal aortic aneurysms. Eur J Vasc Endovasc Surg.

[24] Verhoeven EL, Katsargyris A, Bekkema F (2015). Ten-year experience with endovascular repair of thoracoabdominal aortic aneurysms: results from 166 consecutive patients. Eur J Vasc Endovasc Surg.

[25] Browne TF, Hartley D, Purchas S, Rosenberg M, Van Schie G, Lawrence-Brown M (1999). A fenestrated covered suprarenal aortic stent. Eur J Vasc Endovasc Surg.

[26] Eagleton MJ, Follansbee M, Wolski K, Mastracci T, Kuramochi Y (2016). Fenestrated and branched endovascular aneurysm repair outcomes for type II and III thoracoabdominal aortic aneurysms. J Vasc Surg.

[27] Mastracci TM, Eagleton MJ, Kuramochi Y, Bathurst S, Wolski K (2015). Twelve-year results of fenestrated endografts for juxtarenal and group IV thoracoabdominal aneurysms. J Vasc Surg.

[28] Oderich GS, Greenberg RK, Farber M, Zenith Fenestrated Study Investigators (2014). Results of the United States multicenter prospective study evaluating the Zenith fenestrated endovascular graft for treatment of juxtarenal abdominal aortic aneurysms. J Vasc Surg.

[29] Sweet MP, Hiramoto JS, Park KH, Reilly LM, Chuter TA (2009). A standardized multi-branched thoracoabdominal stent-graft for endovascular aneurysm repair. J Endovasc Ther.

[30] Bakoyiannis CN, Economopoulos KP, Georgopoulos S (2010). Fenestrated and branched endografts for the treatment of thoracoabdominal aortic aneurysms: a systematic review. J Endovasc Ther.

[31] Schanzer A, Baril D, Robinson WP, Simons JP, Aiello FA, Messina LM (2015). Developing a complex endovascular fenestrated and branched aortic program. J Vasc Surg.

[32] Marzelle J, Presles E, Becquemin JP (2015). Results and factors affecting early outcome of fenestrated and/or branched stent grafts for aortic aneurysms. Ann Surg.

[33] Chang CK, Chuter TA, Niemann CU (2009). Systemic inflammation, coagulopathy, and acute renal insufficiency following endovascular thoracoabdominal aortic aneurysm repair. J Vasc Surg.

[34] Haulon S, D’Elia P, O’Brien N (2010). Endovascular repair of thoracoabdominal aortic aneurysms. Eur J Vasc Endovasc Surg.

[35] Chuter TA, Rapp JH, Hiramoto JS, Schneider DB, Howell B, Reilly LM (2008). Endovascular treatment of thoracoabdominal aortic aneurysms. J Vasc Surg.

[36] Harrison SC, Agu O, Harris PL, Ivancev K (2012). Elective sac perfusion to reduce the risk of neurologic events following endovascular repair of thoracoabdominal aneurysms. J Vasc Surg.

[37] Sultan S, Hynes N, Kavanagh EP, Diethrich EB (2014). How does the multilayer flow modulator work? The science behind the technical innovation. J Endovasc Ther.

[38] Thubrikar MJ, Robicsek F, Labrosse M, Chervenkoff V, Fowler BL (2003). Effect of thrombus on abdominal aortic aneurysm wall dilation and stress. J Cardiovasc Surg.

[39] Sultan S, Kavanagh EP, Bonneau M, Kang C, Alves A, Hynes NM (2016). Kinetics of endothelialization of the multilayer flow modulator and single-layer arterial stents. Vascular.

[40] Vaislic CD, Fabiani JN, Chocron S, STRATO Investigators Group (2014). One-year outcomes following repair of thoracoabdominal aneurysms with the multilayer flow modulator: report from the STRATO trial. J Endovasc Ther.

[41] Vaislic CD, Fabiani J, Chocron S, STRATO Investigators Group (2016). Three-year outcomes with the Multilayer Flow Modulator for repair of thoracoabdominal aneurysms: a follow-up report from the STRATO trial. J Endovasc Ther.

[42] Benjelloun A, Henry M, Taberkant M, Berrado A, Houati RE, Semlali A (2016). Multilayer flow modulator treatment of abdominal and thoracoabdominal aortic aneurysms with side branch coverage: outcomes from a prospective single-center moroccan registry. J Endovasc Ther.

[43] Sultan S, Hynes N (2013). One-year results of the multilayer flow modulator stent in the management of thoracoabdominal aortic aneurysms and type B dissections. J Endovasc Ther.

[44] Sultan S, Sultan M, Hynes N (2014). Early mid-term results of the first 103 cases of multilayer flow modulator stent done under indication for use in the management of thoracoabdominal aortic pathology from the independent global MFM registry. J Cardiovasc Surg.

[45] Sultan S, Hynes N, Sultan M, MFM Collaborators (2014). When not to implant the multilayer flow modulator: lessons learned from application outside the indications for use in patients with thoracoabdominal pathologies. J Endovasc Ther.

[46] Hynes N, Sultan S, Elhelali A (2016). Systematic Review and Patient-Level Meta-analysis of the Streamliner Multilayer Flow Modulator in the Management of Complex Thoracoabdominal Aortic Pathology. J Endovasc Ther.

